# A CARE-compliant article: a case report of pleural empyema secondary to *Klebsiella pneumoniae* liver abscess with a hepatopleural fistula

**DOI:** 10.1097/MD.0000000000019869

**Published:** 2020-04-17

**Authors:** Eun Ji Lee, Kyung Hee Lee, Jun Ho Kim, Yong Sun Jeon, Jung Soo Kim

**Affiliations:** a Department of Radiology; b Department of Internal Medicine, Inha University Hospital, Inha University College of Medicine, Jung-gu, Incheon, South Korea.

**Keywords:** empyema, *Klebsiella pneumoniae*, liver abscess, multiplanar reconstruction

## Abstract

**Introduction:**

*Klebsiella pneumoniae* liver abscess (KPLA) is often associated with accompanying metastatic complications such as septic pulmonary embolism, brain abscess, and endophthalmitis. Pleural empyema secondary to a KPLA is a very unusual finding, made even more rare with the presence of a hepatopleural fistula.

**Patient concerns:**

An 81-year-old woman presented with aggravated dyspnea.

**Diagnosis:**

The patient was diagnosed with KPLA with empyema through computed tomography (CT) scan findings and pleural fluid culture.

**Interventions:**

The empyema was drained by thoracostomy, and treatment with empirical antibiotics was initiated. After early removal of the chest tube, the liver abscess as well as the empyema increased. An additional liver abscess drainage procedure was performed.

**Outcomes:**

The fever resolved and dyspnea improved following drainage of effusion. Three days later, the follow-up chest radiograph showed decreased pleural effusion.

**Conclusion:**

Pleural empyema is a rare but fatal complication secondary to KPLA. Additionally, the discovery of a hepatopleural fistula on a CT scan (multiplanar reconstruction image) made this case even more rare. Both, the liver abscess and pleural empyema, were effectively drained through the fistula tract with drainage procedure, thoracostomy, and additional liver abscess drainage. Prompt diagnostic evaluation, using an imaging modality such as CT, and early drainage management with intravenous antibiotics can improve clinical outcome.

## 1 Introduction

*Klebsiella pneumoniae* has been the most commonly isolated pathogen from a pyogenic liver abscess in Asia, and in recent times it is 1 of the common causes of pyogenic liver abscess in Western countries, such as the United States.^[1]^

Patients with a *Klebsiella pneumoniae* liver abscess (KPLA) have often suffered from accompanying metastatic complications.^[1–3]^ In most cases with concurrent KPLA and metastatic *Klebsiella pneumoniae* infections in the lungs, a septic pulmonary embolism has been reported.^[1–3]^ There are very few reports on cases of KPLA, in which pleural empyema is caused through a direct invasion of the thoraces.^[4,5]^ Diagnosing a pleural empyema resulting from a hepatopleural fistula via computed tomography (CT) imaging is extremely rare. Here, we report a unique case of pleural empyema secondary to KPLA with a hepatopleural fistula, that was treated with drainage of the pleural empyema and liver abscess.

## 2 Case report

An 81-year-old woman, with a past medical history of hypertension, visited the emergency department complaining of dyspnea. Her vital signs were as follows: pulse, 69 beats/min; blood pressure, 140/88 mm Hg; respiratory rate, 28 breaths/min; temperature, 35.2°C. On physical examination, she presented decreased breath sounds in the right lung. Initial arterial blood gas analysis with the patient breathing ambient air showed a pH of 7.26, a PCO_2_ of 52.5 mm Hg, a PO_2_ of 74.7 mm Hg, and an oxygen saturation of 92%. Blood test results were as follows: leukocytosis, 14,240/mm^[3]^ (neutrophil 88.6%); C-reactive protein, 12.64 mg/dL; erythrocyte sedimentation rate, 77 mm/h; aspartate aminotransferase, 24 IU/L; alanine aminotransferase, 24 IU/L; total bilirubin, 0.6 mg/dL; and alkaline phosphatase, 89 IU/L.

**Figure 1:**
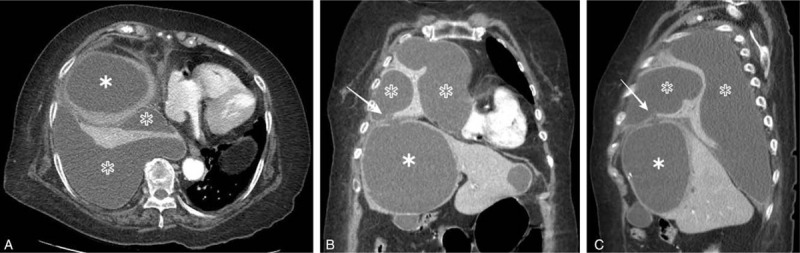
(A): Axial scan (B): Coronal scan (C): Sagittal scan. The initial axial CT scan (A) showed a large abscess (asterisk) that occupied most of the right lobe of the liver at hepatic S4 region which extended into the right pleural space. Coronal, sagittal reformatted images (B, C) from the CT data showed the hepatopleural fistula (arrow). There was a large amount of right empyema (empty asterisk) with diffuse pleural thickening, passive atelectasis of the right lung, and mediastinal shifting to left.

A chest radiograph showed a right massive pleural effusion in the thorax and a mediastinal shift towards the left side. The initial contrast-enhanced chest CT scan showed a large abscess measuring 12.4 × 9.0 × 11.3 cm that occupied most of the right lobe of the liver in the hepatic medial (S4) segment and extended into the right pleural space, revealing a hepatopleural fistula (Fig. 1). There was a large amount of right loculated pleural effusion with diffuse pleural thickening, passive atelectasis of the right lung, and mediastinal shift to the left.

To treat the respiratory failure, an endotracheal intubation was performed and a 12-French chest tube for drainage was inserted into the right thorax. The drained pleural fluid was cloudy and yellow in color. A pleural fluid analysis revealed that the drained fluid was exudative in nature and the subsequent pleural fluid culture was positive for *Klebsiella pneumoniae.* We initiated parenteral antibiotic treatment with piperacillin and levofloxacin. After six days of treatment, the patient's breathing improved and follow up blood tests revealed decreased C-reactive protein level (1.17 mg/dL) and normal erythrocyte sedimentation rate level (18 mm/h). She was transferred from the intensive care unit to the general ward.

**Figure 2:**
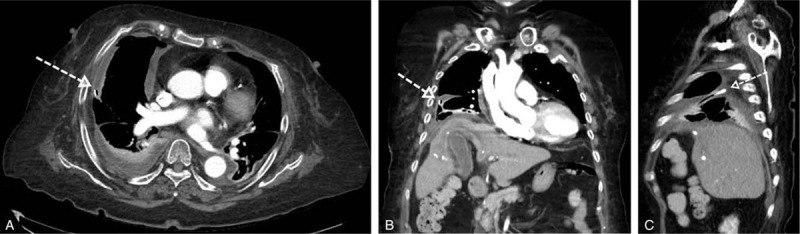
(A): Axial scan (B): Coronal scan (C): Sagittal scan. Right pleural empyema markedly decreased on axial, sagittal scan (A, C) of the follow-up CT. Also, the liver abscess markedly decreased, and curvilinear low attenuated lesion in the right lobe of the liver was still visible on the coronal CT scan (B). There is a chest tube in the right pleural space (empty arrow).

Fifteen days after hospitalization, a follow-up CT scan revealed that both the pleural empyema and liver abscess had markedly decreased (Fig. 2). The chest tube was removed 28 days after hospitalization. Five days after removal of the chest tube, the patient again presented with dyspnea and fever. A subsequent CT scan revealed aggravation of the liver abscess with empyema. A drain was inserted into the liver abscess. Blood cultures of the liver abscess were negative.

Three days after drainage of the liver abscess, a chest radiograph showed that the right pleural effusion had decreased, and the patient's fever and dyspnea improved.

## 3 Discussion

Pyogenic liver abscesses seldom extend into the pleural space, because the diaphragm is a tough membranous barrier.^[6]^ As a result, cases of pleural empyema secondary to liver abscess have rarely been reported in the literature.^[4]^ Therefore, the present case of KPLA resulting in pleural empyema is a rare finding.

Only a few cases of pleural empyema secondary to KPLA have been reported^[4,5,7,8]^ (Table 1). The median age of these patients was 55.5 years (range, 44–65 years). Two patients not only had pleural empyema but also purulent pericarditis.^[7,8]^ The other two cases were empyema caused by a transdiaphragmatic extension of a liver abscess, as in our case.^[4,5]^ Our case is the first pleural empyema to be secondary to KPLA, and to identify a hepatopleural fistula on a CT scan. Multidetector CT (MDCT) clearly identified a hepatopleural fistula tract between the pleural space and the liver abscess through a multiplanar reconstruction image. MDCT allows thinner collimation and enables the acquisition of high-resolution volume data sets. Multiplanar reconstruction facilitates a definitive diagnosis, as seen in our case.^[9]^

**Table 1:**
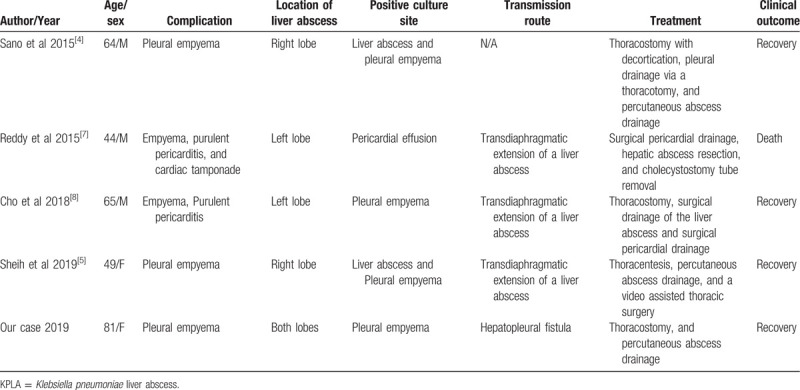
The cases of pleural empyema secondary to KPLA.

In the 2 cases with only pleural empyema (Table 1), there was a liver abscess in the right lobe.^[4,5]^ In our case report, although the woman had an abscess in both lobes, the main abscess with the hepatopleural fistula was in the right lobe of the liver. The other 2 cases which involved the left lobe resulted in pericarditis.^[7,8]^ It is suggested that the liver abscess in the left lobe is more likely to cause pericarditis as well as empyema through transdiaphragmatic extension than the liver abscess in the right lobe. This is thought to be related to where the heart is located primarily on the left thorax. These findings suggest that the development of empyema and pericarditis depend on the predominant KPLA location.

Both pleural fluid and liver abscess culture were positive in 2 of the patients with pleural empyema.^[4,5]^ In our case, *Klebsiella pneumoniae* was isolated only from the pleural fluid culture, and no microorganisms were isolated from the liver abscess. This might be due to the use of antibiotic treatment prior to the cultivation test.

In the patients with KPLA, the most common site of metastasis was the lung;^[1,2]^ septic pulmonary embolism has also been reported.^[1,2]^ Only a few cases of KPLA have been shown to directly invade the thoraces, as reported in our case. Recently, the capsular antigens K1 and K2, along with the presence of virulence gene, such as *rmpA*, have been regarded as important virulent factors of *Klebsiella pneumoniae* and contribute to the development of invasive disease.^[1]^ The K1 strain has been reported to be highly virulent on the account of hypermucoviscosity, which is related to high serum resistance, high-level resistance to phagocytosis, and complement deposition.^[8]^ Although these highly virulent strains of *Klebsiella pneumoniae* could be associated with local invasiveness, the exact mechanism of how this occurs is unknown. All previously reported cases,^[4,5,7,8]^ including our case, did not serotype *Klebsiella pneumoniae* isolates. Further investigation of the virulence factors related to transdiaphragmatic invasion in patients with KPLA is required.

The mortality rate of KPLA ranges from 3% to 17.1%.^[1]^ The metastatic complications in patients with KPLA are usually severe, associated with a poor prognosis, and high mortality rate.^[1]^ Chang et al suggested that immediate drainage of the liver abscess can decrease the spread of metastatic infection, because it reduces the incidence of thrombophlebitis in the hepatic vein.^[1]^ The treatments for the KPLA related empyema,^[4,5,7,8]^ as reported in Table 1, were as follows: thoracostomy in 2 cases,^[4,8]^ thoracentesis in 1 case,^[5]^ additional surgical decortication and drainage of empyema in two cases,^[4,5]^ percutaneous hepatic abscess drainage in 2 cases,^[4,5]^ surgical abscess drainage in 1 case,^[8]^ and surgical hepatic abscess resection in 1 case.^[7]^ All patients were treated with antibiotics and underwent a drainage procedure. In the other cases, the liver abscess was drained first, but we performed a thoracostomy as an initial drainage procedure due to the patient's acute respiratory failure. Subsequently, both the empyema and the liver abscess decreased through thoracostomy and empirical antibiotics. However, both empyema and the liver abscess increased despite maintaining antibiotic treatment on follow-up CT. We speculated that the drainage of the liver abscess entered through the fistula tract during the thoracostomy, which was part of the empyema treatment. Percutaneous thoracostomy through the 7th intercostal space, with an excellent exposure of both the diaphragm and cavity of the hepatic abscess, has been described.^[10]^ In our case, only a thoracostomy provided effective pus drainage of both the thoracic cavity and the liver, which were communicated through the fistula tract identified on the multiplanar reconstruction CT imaging.

However, the empyema and liver abscess increased after removal of the chest tube. Finally, both liver abscess and empyema resolved through additional hepatic abscess drainage. Liver abscess and empyema recurrence may be due to insufficient drainage as a result of early removal of the chest tube.

In conclusion, empyema is a rare but fatal complication secondary to KPLA. To our knowledge this case report is the first to describe pleural empyema and KPLA, which were identified through a hepatopleural fistula on MDCT. Both spaces were efficiently drained via only a thoracostomy and additional liver abscess drainage. If a patient with KPLA presents with dyspnea or pleural effusion, we should keep in mind that pleural empyema can be caused by a liver abscess. Early diagnostic evaluation, using MDCT, and immediate treatment, such as drainage combined with intravenous antibiotics can improve clinical outcome. Establishing standard treatment guideline and further investigation of the virulence factors in patients with empyema secondary to KPLA is required.

## Author contributions

**Data curation:** Eun Ji Lee, Jun Ho Kim

**Supervision:** Kyung Hee Lee

**Writing – original draft:** Eun Ji Lee, Jun Ho Kim

**Writing – review and editing:** Yong Sun Jeon, Jung Soo Kim
